# Optimizing hybrid ensemble feature selection strategies for transcriptomic biomarker discovery in complex diseases

**DOI:** 10.1093/nargab/lqae079

**Published:** 2024-07-11

**Authors:** Elsa Claude, Mickaël Leclercq, Patricia Thébault, Arnaud Droit, Raluca Uricaru

**Affiliations:** Univ. Bordeaux, CNRS, Bordeaux INP, LaBRI, UMR 5800, F-33400 Talence, France; Molecular Medicine Department, CHU de Québec Research Center, Université Laval, Québec, QC, Canada; Molecular Medicine Department, CHU de Québec Research Center, Université Laval, Québec, QC, Canada; Univ. Bordeaux, CNRS, Bordeaux INP, LaBRI, UMR 5800, F-33400 Talence, France; Molecular Medicine Department, CHU de Québec Research Center, Université Laval, Québec, QC, Canada; Univ. Bordeaux, CNRS, Bordeaux INP, LaBRI, UMR 5800, F-33400 Talence, France

## Abstract

Biomedical research takes advantage of omic data, such as transcriptomics, to unravel the complexity of diseases. A conventional strategy identifies transcriptomic biomarkers characterized by expression patterns associated with a phenotype by relying on feature selection approaches. Hybrid ensemble feature selection (HEFS) has become increasingly popular as it ensures robustness of the selected features by performing data and functional perturbations. However, it remains difficult to make the best suited choices at each step when designing such approaches. We conducted an extensive analysis of four possible HEFS scenarios for the identification of Stage IV colorectal, Stage I kidney and lung and Stage III endometrial cancer biomarkers from transcriptomic data. These scenarios investigate the use of two types of feature reduction by filters (differentially expressed genes and variance) conjointly with two types of resampling strategies (repeated holdout by distribution-balanced stratified and random stratified) for downstream feature selection through an aggregation of thousands of wrapped machine learning models. Based on our results, we emphasize the advantages of using HEFS approaches to identify complex disease biomarkers, given their ability to produce generalizable and stable results to both data and functional perturbations. Finally, we highlight critical issues that need to be considered in the design of such strategies.

## Graphical Abstract

**Graphical Abstract:**
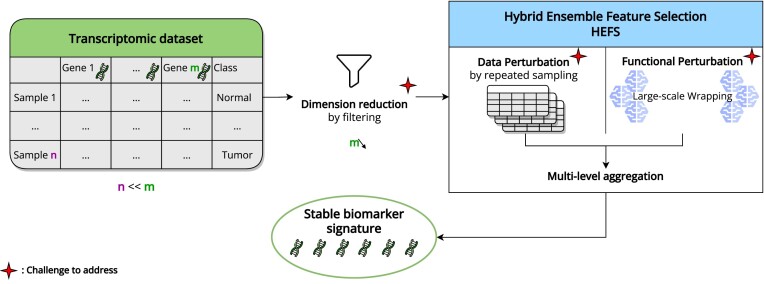

## Introduction

The development of new sequencing methods to generate data at different biological levels, also known as omics (genomics, transcriptomics, etc.), has led to significant advances in medical research. Over the years, this variety of data have helped to better understand complex diseases such as cancer, which remains one of the leading causes of death worldwide. Numerous research studies have highlighted the importance of predicting the diagnosis of cancer patients based on biomarker signatures, defined as a set of biological variables that can discriminate between groups of patients (1). The identification of such a signature, which is performed by using one (2) or several types of omics (3) in bulk or in single-cell context (4), remains a recurring challenge.

In the last two decades, feature selection (FS) strategies, and more specifically those based on machine learning (ML) techniques have been widely developed (5) and applied to omics data to identify potential biomarkers for cancer stage characterization (6,7) or patient prognosis (8,9). Omics data induce challenges such as a large number of features (*i.e*. genes) versus a limited number of observations (samples). This well known problem, known as *the curse of dimensionality*, requires the implementation of strategies to *a priori* reduce the number of features (10).

Moreover, the highly variable resulting feature subsets have motivated the research community to apply and develop ensemble feature selection (EFS) approaches to integrate the various FS methods and produce robust signatures. For this purpose, EFS capitalizes on perturbations of the data (homogeneous) or on functional perturbations (heterogeneous) at the algorithm end of the process, while designing an aggregation step that stabilizes the feature subset result (11). The data perturbation procedure applies an unique FS algorithm on different subsets of a dataset before performing an aggregation, while the functional perturbation process carries different FS methods on the same dataset ahead of the aggregation (11). Regarding the FS method, one can choose from three categories: filter, wrapper and embedded (12). Filters rely on the general characteristics of the data without being dependent on a particular learning method (13). Wrappers and embedded methods on the other hand, inherently build upon the learning process. The former assess the pertinence of a feature subset by evaluating the performance of an associated ML model trained with these features. The latter, include ML techniques for which feature selection is performed intrinsically within the training process (i.e. weight optimization in neural networks or node definition in decision trees) (14).

In this respect, recent studies encourage researchers to develop hybrid EFS (HEFS) approaches that combine variability at the data and the algorithm endpoints so as to benefit from both homogeneous and heterogeneous notions (15,16) in order to increase the stability of the result. Indeed, such approaches should yield more robust conclusions that are independent of a specific input data subset or FS method. Nevertheless, designing HEFS solutions remains challenging as it requires various decisions to be made including the choice of a sampling technique, the number and types of FS methods to use, their tuning, and the aggregation process to apply in the end.

Traditionally, ML methods employ a conventional, random-based sampling strategy when performing the training step (15,17). However, in presence of imbalanced data there is a potential benefit in opting for stratified sampling. This type of approach should ensure a more representative selection of input classes, mitigating the risk of overfitting to a dominant class (18). Moreover, when handling biological datasets and particularly in cancer research, acknowledging intra-class heterogeneity is essential to address inter-patient variability. Recognizing this variability is critical due to the emergence of diverse clonal populations converging into a unified phenotype, a well-documented situation in cancer (19).

An increasing effort has recently been made to evaluate and identify the most appropriate dimension reduction method given a type of data. For example, a study by Bommert *et al.* (20) on gene expression survival data identified the variance filter as being the best among 15 filtering methods when considering the accuracy of a unique downstream ML model, the runtime and the stability of the selection. In cancer diagnosis based on transcriptomic data, studies often rely on differentially expressed genes analysis (DEG) as a preliminary step before engaging in machine learning processing to identify biomarkers (21–24). Regarding the wrapping methods, they result in a unique, finely tuned ML model. However, different hyper-parameter configurations may lead to multiple models showing good performances (though not necessarily the best), with different signature compositions. This suggests that intra-algorithm variability should be considered when developing an ensemble approach.

Additionally, HEFS strategies should benefit from the integration of various FS methods of different types such as filters and embedded ones as in (16) or filters and wrappers as in (25) or filters, wrappers and embedded methods as in (21). This would allow capitalizing on their respective strengths and weaknesses. Indeed, compared to wrapping or embedded methods, filters disregard the impact of the selected feature subset on downstream ML prediction and can be employed as dimension reducers (26). Wrappers alone, on the other hand, can be highly computationally expensive (27) and could benefit from a dimensional reduction. Additionally, wrappers can be designed to scale to multiple types of classifiers in order to explore their intra-algorithm variability. Finally, embedded methods depend on a unique learning algorithm and therefore cannot scale to multiple ML algorithms.

To date, a comprehensive investigation of the impact of various design choices in formulating a HEFS strategy for biomarker selection from RNA-seq gene expression data is lacking in the scientific literature. There is indeed a notable absence of scientific evaluations regarding the relevance of integrating dimension reduction filters in conjunction with a judicious sampling methodology and a rich set of wrappers with different hyper-parameter configurations.

To fill this gap, in this work we propose an extensive study comparing four HEFS scenarios for the biomarker identification from RNA-seq data of Stage IV colon and rectum adenocarcinoma, *i.e*. colorectal cancer (CRC), with respect to normal samples. Moreover, we applied our HEFS approach for the identification of Stage I Kidney Renal Clear Cell Carcinoma (KIRC), of Lung Adenocarcinoma (LUAD) and of Stage III Uterine Corpus Endometrial Carcinoma (UCEC) biomarkers. In this manuscript we first present the datasets and the setup of our HEFS scenarios including the investigation of two sampling approaches, the filtering considerations, the choice of the wrapping strategy, the hyper-parameter tuning of several ML methods for the computation of thousands of models, and the multi-level aggregation of the results. Secondly, we provide extensive results and their analysis and comparison in the computation of stable signatures for CRC, using a standard mode of our HEFS protocol. We further evaluate the robustness of the results obtained with the various HEFS scenarios based on an external evaluation CRC dataset. Finally, we studied the ability of our HEFS approach to generalize to other types of cancer with a light mode protocol, which involves a less stringent early dimensional reduction by differential expression analysis (DEG) and the training of fewer wrappers. In a third section we address and propose viable solutions regarding the manifold challenges that arise during the computation of consensus in biomarker signatures with HEFS strategies. We then conclude and expose potential perspectives of the work presented in this study.

## Materials and methods

### Data collection and processing

*Train dataset: TCGA CRC*. Our approach was experimented on a dataset from the Cancer Genome Atlas program (TCGA) database. Colon and rectum adenocarcinoma (CRC) data were obtained from the Genomic Data Commons Data (GDC) portal using the TCGAbiolinks R package (28,29). RNA-seq read counts of the Colon adenocarcinoma (COAD) and Rectum adenocarcinoma (READ) cohorts were retrieved (30), for a total of 462 and 171 patients respectively before filtering. Data were divided into two classes of samples labeled according to the American Joint Committee on Cancer (AJCC) stage score: 1. Normal, solid tissue normal and 2. Stage IV, primary tumor. Clinical data were used to map tumor samples to their corresponding AJCC score such that all Stage IV (IV, IVa, IVb, IVc) samples were mapped under the Stage IV label. All solid tissue normal samples were gathered in a Normal class. Biospecimen data were used to filter out samples having <70% of tumor nuclei as well as FFPE samples and to manage patient duplicates and retrieve batch information.

*Evaluation dataset: GSE50760*. To evaluate the relevance of signatures obtained on the dataset described above, we selected an additional, independent dataset, i.e. GSE50760 from the GEO database (31). This dataset gathers RNA-seq data from 18 patients having Stage IV CRC and from which 3 types of samples were collected: solid tissue normal, primary tumor and metastases. As we were interested in characterizing the Stage IV CRC primary tumor we only retrieved the first and second type of samples. We labeled the first as Normal class and the second as Stage IV class. To obtain read counts like for the TCGA CRC dataset, a similar pre-processing as the one performed by the GDC portal was used. First, a quality control was applied on the raw data using the FastQC software and then, a mapping of the sampling reads was performed against the hg38 genome using the STAR software and with the –quantMode GeneCounts parameter (32).

*Additional TCGA datasets: KIRC, LUAD and UCEC*. The same data collection and processing procedure as detailed above was applied on three additional TCGA datasets corresponding to Kidney Renal Clear Cell Carcinoma (KIRC), Lung Adenocarcinoma (LUAD) and Uterine Corpus Endometrial Carcinoma (UCEC). In order to compute biomarkers for Stage I versus normal, for both KIRC and LUAD, AJCC Stage I annotated primary tumor samples were retrieved as the Stage I class and Solid Tissue Normal from the associated cohort as the Normal class. For the UCEC analysis, the Stage III class is composed of the AJCC Stage III primary tumor samples and the Normal class is based on Solid Tissue Normal ones.

### HEFS approach

*Overview*. To investigate the impact of filtering, sampling and aggregation stages in the context of biomarker identification from transcriptomic data, four scenarios have been designed within a Hybrid Ensemble Feature Selection (HEFS) framework. First, a dimension reduction has been performed by applying a gene filtering stage based on either a differentially expressed genes analysis (DEG), or an analysis of variance (Var) (see *Filtering step* section). Second, data perturbation has been considered by experimenting a random stratified sampling (R-S) method versus a distribution-balanced stratified sampling one (DB-S) (see *Dataset sampling strategies* section). Third, these four scenarios have been submitted to a large-scale wrapping process that trains thousands of wrappers from eight widely used classifiers having various hyper-parameter configurations (see *Wrapping and Aggregation* section). Finally, we proposed generic rules to combine the results through an aggregation strategy. The idea behind our aggregation method is manifold: to assess the effects of both the preliminary filtering stages and the two types of samplings, as well as to take advantage of the diversity and complexity of diverse ML methods by testing multiple combinations of hyperparameters (see *Wrapping and Aggregation* section).

*Filtering step*. From the input dataset and after removing protein coding genes and ncRNAs with less than 10 mapped reads across all samples, two types of filters were applied: one based on a differentially expressed genes analysis (DEG) and the other based on a variance (Var) analysis.

The DEG analysis, performed using the DESeq2 R package, takes into account possible batch effects and the associated labels of the data (Normal or Stage IV). Data was then transformed with the classical variance stabilizing transformation method. Finally, we retained protein coding genes and ncRNAs with a fold change of at least 2 and an adjusted *P*-value <0.001 or 0.05 for the HEFS standard mode or light mode, respectively (33–35).

The variance filter was performed using the edgeR package to normalize the data into log of count per millions followed by a removal of the batch effect using the limma R package (36–38). Finally, the cola R package's adjust_matrix function selected the 10% most varying variables (i.e. genes) across all samples without considering the conditions beforehand (39).

**Figure 1.**
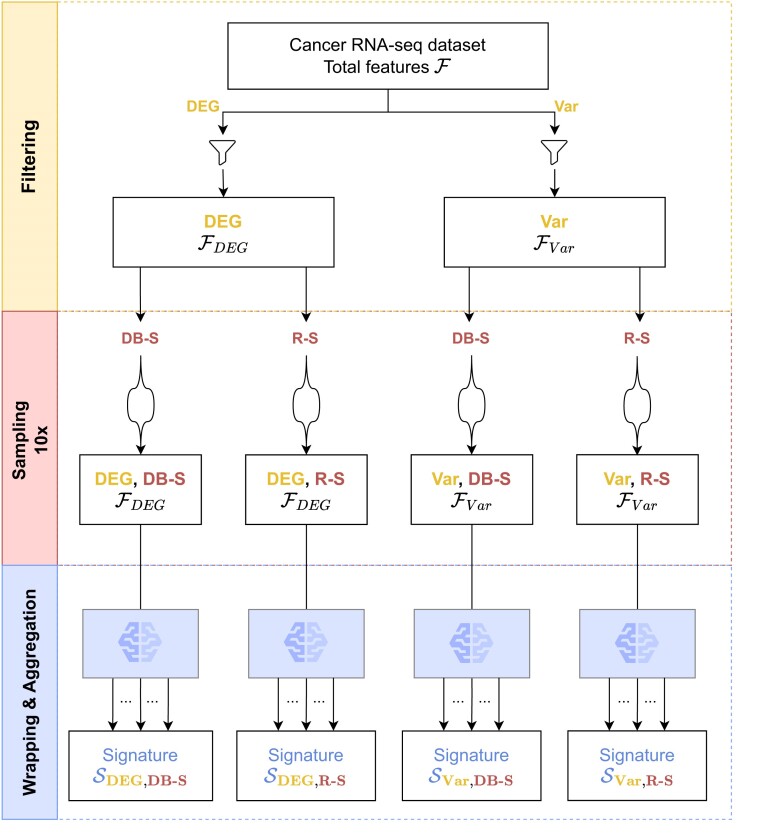
Overview of the multiple hybrid ensemble feature selection (HEFS) scenarios, from filtering and sampling considerations to feature selection with wrapper machine learning models. The four scenarios were applied in the context of the identification of biomarkers from cancer RNA-seq datasets. Filtering applied to the data is either based on differentially expressed genes analysis (DEG) or on variance analysis (Var). A repeated holdout sampling step to compute 10 different pairs of training and test sets for the feature selection step by wrapping is performed. It is either done by random stratified sampling (R-S) or distribution-balanced stratified sampling (DB-S). In the end, each scenario performs multiple wrappings followed by an aggregation step.

The above threshold values have been chosen in order to reduce the number of variables for the subsequent wrapping process, which is demanding in terms of computational resources. Having applied these filters on an input dataset, two filtered datasets that are labeled respectively by the prefixes DEG and Var were computed. (Figure 1, filtering panel).

*Dataset sampling strategies*. Here we implemented and tested two types of sampling producing pairs of training and test sets. Both perform repeated holdouts as they split the input in$\;n$ (here $n = 10$), different pairs of training sets (2/3) and test sets (1/3). The first version of sampling is a variant of a stratified random sampling (R-S) as introduced in (40) that preserves the initial distribution between the classes. The second sampling strategy is a variant of distribution-balanced stratified cross-validation (DBSCV) as presented in (41,42) and is referred to as DB-S in our study. It takes into account the intra-class variability by defining intra-class clusters with the aim, in our case, to preserve inter-patient heterogeneity. The clusters were computed by applying a hierarchical clustering using the Euclidean distance and Ward's method for the linkage. A Silhouette technique was then applied to automatically define the appropriate number of clusters (43), which allowed us to associate sample IDs to those newly defined clusters. We then performed a two-level stratified sampling to ensure that both inter- and intra-class distributions are respected when defining 10 pairs of training and test sets.

*Evaluation metrics*. Our HEFS scenarios include a wrapping step where thousands of models are trained. The performance of the models was evaluated by the Matthews Correlation Coefficient (MCC), which, compared to the accuracy or the F-1 score, is known to be a good compromise in the case of imbalanced data (44).

In order to measure the feature stability brought by the various FS methods used, we applied the Nogueira score (45,46). A Nogueira score has an upper bound of 1 and a lower bound of $ - 1/( {p - 1} )$ where $p$ is the number of feature sets to compare. A Nogueira score close to 1 indicates high stability of the compared feature sets while a score close to 0 suggests low stability.

#### Wrapping and aggregation

**Figure 2.**
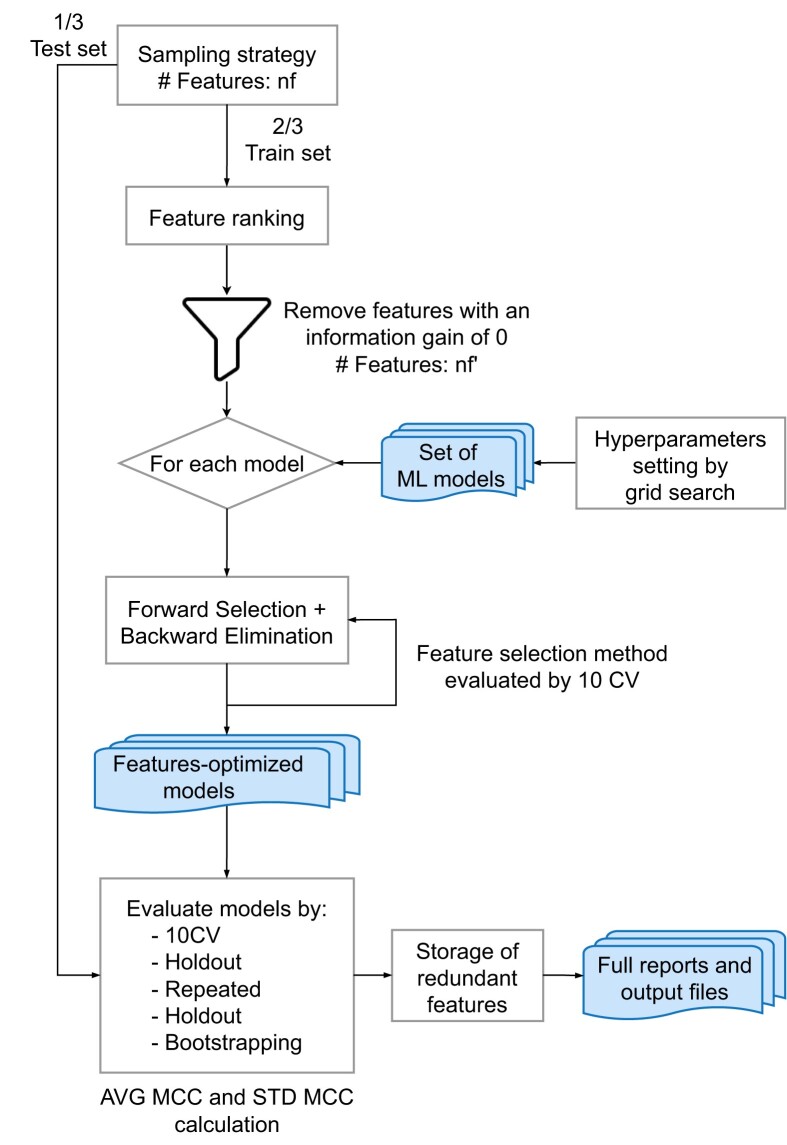
ML training process based on BioDiscML software (Figure adapted from (47)). Input data for a sampling run, composed of patients described by nf features, is split into 1/3 test and 2/3 train sets. The train set undergoes a feature ranking process with the information gain measure, where features having a score of 0 are discarded. The resulting features nf’ are then submitted to a training loop for each model (thousands of model configurations were generated for the 8 types of classifiers). This loop is based on a Forward Stepwise Selection combined to a Backward Stepwise Elimination procedure (see *Optimal feature subset search methods* in *Materials and methods* of (47) for details). Resulting trained models are going through various cross validation steps and for each of them, the average MCC (AVG MCC) and the associated standard deviation of the MCC (STD MCC) are computed. Redundant features, discarded during the training process but having meaningful information, are put aside for further analysis.

*Wrapping and aggregation—wrapping process*. The wrapping process (Figure 2) was carried out based on the BioDiscML software, which is a state-of-the-art, easy-to-use pipeline that is able to perform ML wrapping while keeping a record of all experiments (47). Training phases for each sampling run were time limited and had predefined computational resources. As we intend to produce a stable output from various wrappers, thousands of models were computed with multiple hyper-parameter configurations generated with a grid search approach (Supplementary Table S1). Each training dataset goes through a first FS step based on the information gain score (48), at the end of which features having an information gain score of 0 are discarded. Then, for each model, the training datasets are submitted to a Forward Stepwise Selection and Backward Stepwise Elimination training loop (see (47) for details on the training procedure), a form of Recursive Feature Elimination (RFE) strategy (49) widely used by FS methods applied to biomedical problems. This allows optimization of model performances by computing an MCC score at each iteration and finally selecting a restricted set of high-quality features. Features being redundant of the ones selected in this step were kept for further analysis. Redundancy was evaluated using the Pearson and Spearman correlations, as well as the information gain score. Features having a Pearson's or Spearman's correlation score higher than 0.985 were considered as being redundant. Moreover, given the binary classification context, two features having the same information gain score were also considered to be redundant, as this indicates a similar power of separation between classes.

The models generated by the training loop were further evaluated in order to assess their potential overfitting on the input data. This was done with several cross-validation procedures on the data including the test sets: 10 cross-validation, repeated holdout and bootstrapping (50). For each model, the average MCC (AVG MCC), as well as the associated standard deviation MCC (STD MCC) were computed. An AVG MCC value of 1 along with a STD MCC of 0 indicates a perfect model.

To gather diversity from multiple ML algorithms, as encouraged in functional perturbation based approaches, we selected eight supervised ML methods that are often used in biomedical studies to resolve classification problems (51). These classifiers can be roughly organized in four categories: Bayes, Trees, Lazy and Function based. Among the Bayes probabilistic classifiers, Naive Bayes (52), Bayesian Network (53) and Averaged 1-Dependence Estimators (A1DE) (54) were selected. The first two are often applied on biological data, while the third one was developed to address the attribute-independence issue of the Naive Bayes method. When considering decision Tree-based classifiers (55) that are embedded FS algorithms, we selected C4.5 and Random Forest that are commonly used in a clinical context. We also used Simple Classification And Regression Trees (Simple CART) (55) whose logic is similar to the C4.5 method. We also considered the classical Lazy *k*-Nearest Neighbors kNN classifier (56). Finally, we selected the Support Vector Machine (SVM), a function-based classifier known to give high quality results in a classification context for biomedical problems (57).

*Wrapping and aggregation—aggregation method*. Each step in a feature selection strategy has great influence on the signature composition. The initial filtering step, the sampling, but also the chosen classifiers tuned with various hyperparameter values, all lead to variable signature compositions. Thus, taking advantage of multiple such configurations by aggregating the different signatures they produce into a consensus signature result should increase the stability and generalization of FS results.

**Figure 3.**
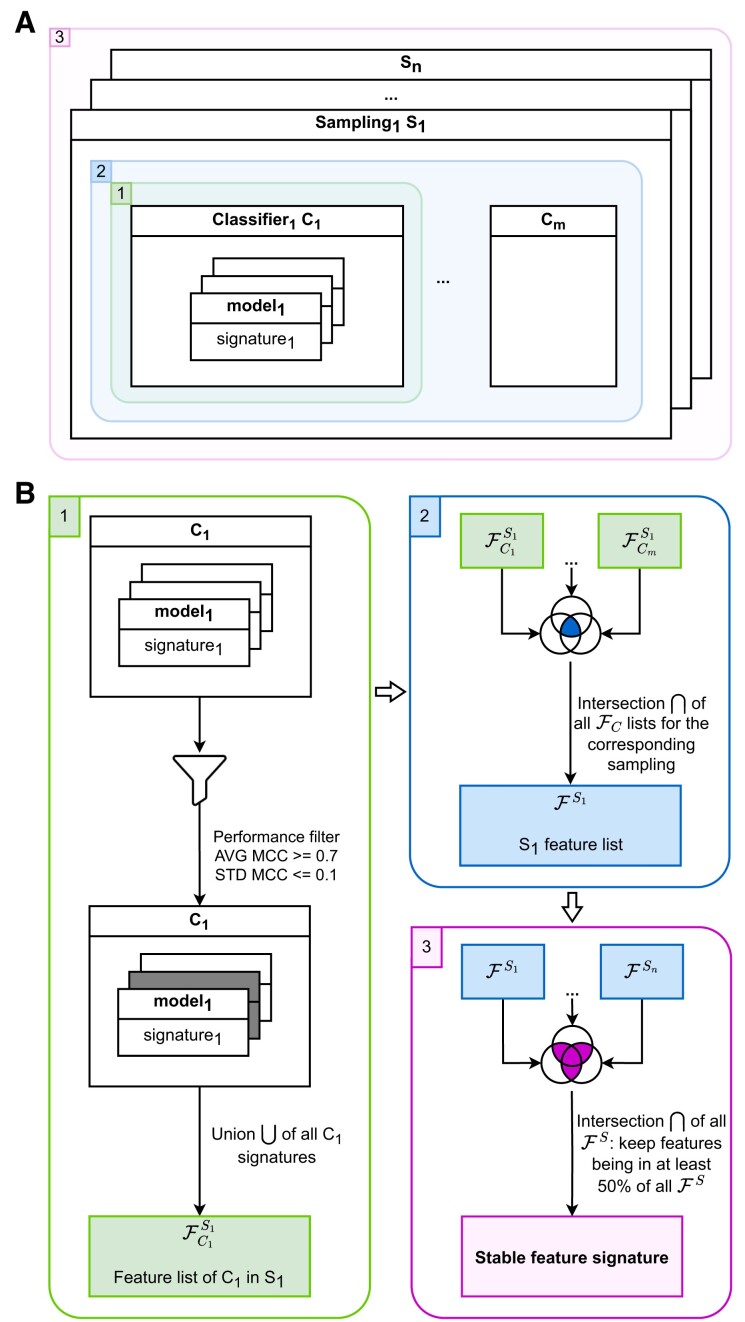
(**A**) Overview of the 3-level aggregation strategy: (A.1) classifying method level (between models of a same classifier and per sampling), (A.2) sampling level (between classifiers for a given sampling), (A.3) global consensus (across samplings). (B.1) At the first level, a classifier-wise process performs the union of the feature signatures computed by ML models having passed the performance filter. (B.2) For the second level, a sampling-wise aggregation is carried out, by computing the intersection per sampling between the lists of features obtained from the various classifiers at the previous level. (B.3) In the end, the sampling feature lists from the previous level are put together in order to obtain a global stable feature signature. This signature will be composed of the features that were included in at least 50% of the samplings.

Here we consider both the functional perturbation complexity (i.e. induced by the type of classifier and the hyper-parameter variability) as well as the data perturbation (i.e. induced by the sampling strategies), by performing a multi-level aggregation. To build a consensus between these various signatures, we considered a three-level integration strategy: integration of model signatures at the classifier level, then at the sampling level among classifiers, and finally across samplings to produce a global consensus (Figure 3A). The first two levels address the functional perturbation problem and the third level takes into account the data perturbation.

For clarity's sake, let us consider $n$ as the number of performed samplings and $m$ the number of type of classifiers. Note that the results in the following of the paper were obtained with $n = 10$ and $m = 8$. A full explanation of our aggregation method follows.

- At the first level of our aggregation process, we performed an integration between the results obtained with models generated for each classifier-sampling combination (Figure 3.B1). First, models were filtered and only those models having an AVG MCC of at least 0.7 and a STD MCC of 0.1 or less were kept. The signatures of the remaining ML models were enriched with the redundant features of their corresponding sampling, which had been put aside during the training process. Then, the union of these enriched signatures was computed for each sampling ${S_i}$ and each classifier ${C_j}$, resulting in $n \times m$ lists of features $F_{{C_j}}^{{S_i}}$.
- Secondly, the aggregation was carried out at the sampling level, thus allowing the integration of the classifier results for a given sampling. In this step, depicted in Figure 3.B2, an intersection of the lists of features obtained for each classifier at the previous step was computed per sampling. This resulted in $n = 10$ lists of features, one per sampling, *i.e*. ${F^{{S_i}}} = \mathop \cap \limits_{j = 1}^m F_{{C_j}}^{{S_i}}$ for a sampling ${S_i}$.
- Finally, the highest level of aggregation was implemented in order to produce a global and stable signature (Figure 3.B3). From the intermediate sampling-wise feature lists (${F^{{S_i}}}$ computed in step 2), a signature was computed by taking those features involved in at least 50% of our $n$ sampling lists.

## Results

### Filtering and sampling for the CRC dataset in the standard mode

Detecting Stage IV is a key task in colorectal cancer research as, while earlier stages have a favorable prognosis when diagnosed, Stage IV (around 21% of patients) presents a negative one (58). After data processing, the TCGA CRC dataset is composed of 138 samples with 87 identified as Stage IV and 51 as Normal, and associated with approximately 40 000 features. After applying in parallel DEG and Var filtering, 5 824 and 4 469 variables are left respectively. These two datasets were further sampled in parallel with the R-S and DB-S strategies in order to obtain the four scenarios: DEG DB-S, DEG R-S, Var DB-S and Var R-S. For Var DB-S, 2 and 3 clusters have been respectively computed for Stage IV data and for Normal data (Supplementary Figures S1 and S2). For DEG DB-S, 2 clusters were defined for both Stage IV and Normal data (Supplementary Figures S3 and S4).

### Model performance on CRC dataset in the standard mode

For each of the four scenarios, around 88 800 models were trained (Table 1).

**Table 1.** Total number of trained models per scenario (rows) and classifier (columns) as well as the total number of trained models per scenario and the total number of models kept after application of the performance filter (AVG MCC of 0.7 or higher and STD MCC of 0.1 or less)

Scenario	A1DE	Bayes network	Naive Bayes	SVM	kNN	C4.5	Random Forest	Simple CART	**Total trained models**	**Efficient models**
DEG DB-S	24 000	1672	30	4521	10 800	8360	19 800	19 800	**88 983**	**85 855**
DEG R-S	24 000	1656	30	4564	10 800	8360	19 800	19 800	**89 010**	**85 808**
Var DB-S	24 000	1629	30	4280	10 800	8360	19 800	19 800	**88 699**	**64 492**
Var R-S	24 000	1595	30	4279	10 800	8360	19 800	19 800	**88 664**	**78 799**

**Figure 4.**
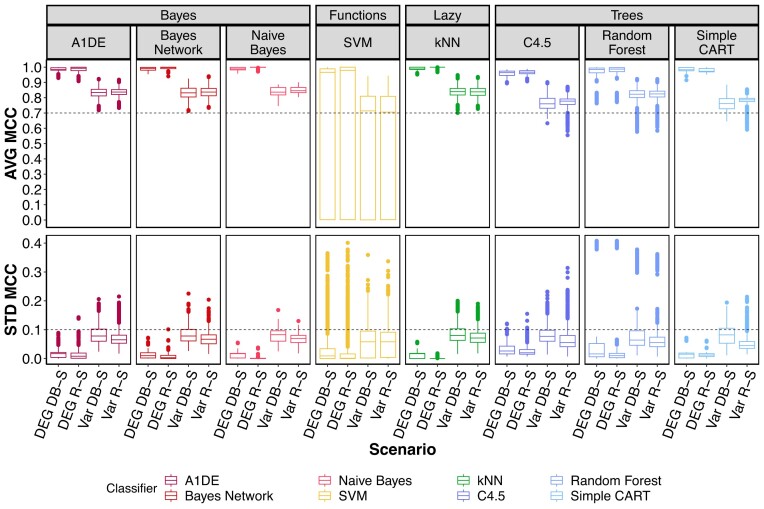
Performance quality metrics computed for four hybrid ensemble features selection (HEFS) scenarios including 10 sampling runs with an average of 88 800 trained models each (for eight different classifiers). The upper panel shows the distribution of Average Matthews Correlation Coefficient (AVG MCC) values, with 1.0 indicating high performing models. The lower panel exhibits the Standard Deviation from the AVG MCC (STD MCC), with 0.0 being the best as it denotes a performance that is equal in all evaluations. In each panel, the black dotted line highlights the threshold value applied to filter out poorly performing models for the following HEFS steps. Thresholds are set to a minimum of 0.7 for AVG MCC and an associated maximal STD MCC of 0.1.

Figure 4 depicts the performance trends (with AVG MCC and STD MCC) of the models according to a classifier and a HEFS scenario.

Interestingly, the two DEG scenarios give trained models with higher scores than the Var scenarios (Wilcoxon tests on the mean of AVG MCC and on the mean of STD MCC, per sampling run; adjusted *P*-value $ \le {10^{ - 2}}$). The range of the scores is the largest for the SVM method, which shows a higher sensitivity to variations of its hyper-parameters, regardless of the scenario. To a lesser extent, Tree-based models show similar behavior.

It should be noted that only those models with scores of 0.7 and above for the AVG MCC and of 0.1 and below for the STD MCC were kept to focus on high quality models. With these thresholds, about 3 000 models (3.4% of the total trained models) were not retained in both DEG scenarios, most of which were computed by SVM and RF methods (Table 1). In the Var scenarios, the effect of this rigorous selection was more severe, with losses of 11.1% and 27.3% for Var R-S and Var DB-S models, respectively.

### Quantitative and qualitative analysis of CRC signatures in the standard mode

**Figure 5.**
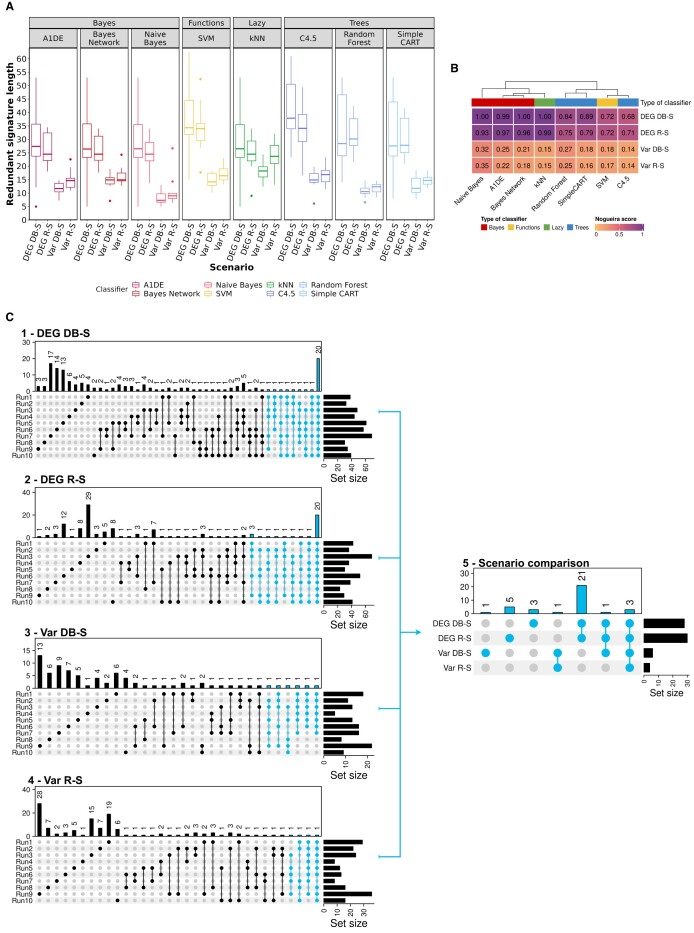
Quantitative analysis of the CRC model signatures. (**A**) Variations in the lengths of the signatures with redundancy. Each boxplot depicts the average signature length for each of the 10 sampling runs. (**B**) Heatmap of the average Nogueira stability scores for all retained models over the 10 sampling runs and for the 4 HEFS scenarios (DEG DB-S, DEG R-S, Var DB-S, Var R-S). (**C**) Feature distribution through all sampling runs for the 4 HEFS scenarios based on the feature lists obtained after the second aggregation step, i.e. sampling-wise (C.1–4) (see *Materials and methods*, *wrapping and aggregation* section). The final upset plot (C.5) corresponds to the comparison of the 4 signatures obtained at the final step of each HEFS scenario aggregation process (in blue in C.1–4).

To go further, in Figure 5 we analyzed the signatures according to the different scenarios and classification methods. The focus was to measure the variation in size of the signatures (Panel A), the stability of the signatures between scenarios and classifiers using the Nogueira metric (Panel B), and the reproducibility with respect to the sampling runs (Panel C).

In Figure 5A we analyze the distribution of signature lengths including redundant information and depending on the employed classifier and scenario. Each boxplot summarizes the average size of all trained models for the 10 sampling runs performed for each scenario. The median signature size varies among classifiers from 24 to 38 for the two DEG scenarios, and from 7 to 17 for the two VAR scenarios, with the exception of the kNN classifier. Noticeably, focusing on the kNN classifier, the signature size distribution is similar for both DEG R-S and Var R-S scenarios with a median size around 24. Overall, we can observe a greater variability in size for DEG versus Var scenarios. This behavior is even more pronounced for the Tree classifiers, where some sampling runs yield signatures larger than 40. Conversely, for both VAR scenarios, signatures remain short regardless of the classifier and of the run iteration.

Now focusing on the composition of the signatures, we used the Nogueira score to evaluate their consistency across models per classifier, sampling run and scenario (Figure 5B). For a given scenario and classifier, the average Nogueira score for the 10 sampling runs are reported in the heatmap. The best scores are given for DEG-based scenarios which means that the features composing the signatures are highly stable through models of the same type of classifier and for each of the 10 sampling runs. Indeed, all Bayes-based models and kNNs have an average Nogueira score above 0.93, while Tree-based and SVMs have a score between 0.68 and 0.89. By contrast, the general trend for VAR-based scenarios is towards lower Nogueira scores which means highly variable signatures.

After following our aggregation process we further address the signature composition variability between sampling runs (Figure 5C.1–4) and between scenarios (Figure 5C.5). For both DEG-based HEFS scenarios, the intersection between the signatures is of 20 variables, thus showing significant stability between sampling runs. On the other hand, for both Var-based HEFS scenarios the intersection is extremely low: 1 for Var R-S and void for Var DB-S. We then performed the final aggregation step (see *Materials and methods*, *wrapping and aggregation* section and Figure 3B3) that gathers for each scenario all features being in at least 50% of the 10 sampling runs feature lists. We obtained 28, 30, 6 and 4 stable variables for DEG DB-S, DEG R-S, Var DB-S and Var R-S scenarios respectively (blue bars in Figure 5C1–4). The intersection of all stable signatures (Figure 5C5) from the four scenarios gives three common features while 29 are specific to DEG scenarios (21 common to both DEG scenarios) and 2 are specific to Var scenarios (1 common to both Var scenarios). The overlap is small due to the size of the four included signatures. However, the smallest variable set from Var scenarios is largely included within the signatures from DEG scenarios.

The 4 signatures have been roughly analyzed from a biological point of view using the DisGeNET database (a database of genes associated with human diseases) through the EnrichR tool (59). Results are shown in Table 2 where genes related to the Colorectal Cancer are in bold. We can observe that the four signatures are well characterized in the CRC context. Noticeably, the KRT80 gene (given by three of the four scenarios) has recently been proposed as an independent prognostic biomarker for CRC based on its role to promote CRC migration and invasion via activation of the AKT pathway (60).

**Table 2.** HEFS scenarios and their associated feature signature

HEFS scenario	Associated signature	Enrichment of Colorectal Cancer annotation (adjusted p-value)
DEG DB-S	**ETV4**, **ESM1**, **OTOP2**, **CDH3**, **SCGN**, **SALL4**, **IL6R**, **SCARA5**, **PVT1**, **ABCA8**, **MRGBP**, **BMP3**, **EIF4E3**, GLP2R, BEST4, FAM135B, CLEC3B, ENPP6, KRT80, CA7, METTL7A, SLC39A10, ELANE, SEMA6A-AS2, SLC51B, SCN9A, MAFG-AS1, TMIGD1	$7.8 \times {10^{ - 2}}$
DEG R-S	**ETV4**, **ESM1**, **OTOP2**, **CDH3**, **SCGN**, **SALL4**, **IL6R**, **SCARA5**, **PVT1**, **ABCA8**, **MRGBP**, **NR3C2**, **CBLN2**, GLP2R, BEST4, FAM135B, CLEC3B, ENPP6, KRT80, CA7, METTL7A, SLC39A10, ELANE, SEMA6A-AS2, SLC51B, SCN9A, MAFG-AS1, PLPP1, NKRF, UGP2	$1.4 \times {10^{ - 1}}$
Var DB-S	**ETV4**, **ESM1**, **OTOP2**, **AJUBA**, KRT80, LINC00974	$5.3 \times {10^{ - 2}}$
Var R-S	**ETV4**, **ESM1**, **OTOP2**, **AJUBA**	$3.4 \times {10^{ - 2}}$

Features highlighted in bold are the one involved in the Colorectal cancer annotation from the DisGeNET database. Third column is the associated adjusted *P*-value performed through the EnrichR web tool.

### Independent CRC signature evaluation

We further evaluated the capacity of the four HEFS signatures to generalize to an external cohort of patients with Stage IV CRC. For this, we trained and optimized ML models on the CRC TCGA data, and then we evaluated the models robustness, and therefore their associated signature on dataset GSE50760.

In order to set up a classical, independent machine learning evaluation, we employed the h2o R package (61) that allows automatic training of multiple ML classifiers with optimization of hyper-parameters by random grid search. First, we considered the two TCGA datasets obtained after the HEFS filtering step performed by DEG and Var (Figure 1). Only variables from the stable signatures of each of the 4 HEFS scenarios were retained: for the DEG scenarios, 28 variables were kept for DB-S and 30 for R-S, while for Var scenarios, six and four variables were kept for DB-S and R-S respectively. We then performed the training of multiple ML models on these four datasets. h2o package was used to train gradient boosting, random forest and linear models. Their hyper-parameters were optimized through an extensive grid search process. The best model was selected by comparing the model performances on a 10 cross-validation evaluation strategy using the associated input TCGA dataset (Table 3). Interestingly, models trained using DEG-based signatures exhibited perfect MCC scores on a 10 cross-validation which may indicate an overfitting. On the other hand, models trained on Var-based signatures were highly performing while having an important standard deviation.

**Table 3.** Machine learning models trained and optimized on four different subsets of the TCGA colorectal cancer dataset and evaluated on the GSE50760 dataset with the Matthew's correlation coefficient

Scenario	Number of features	Best optimized model	Average 10 cross-validation MCC on TCGA	MCC score on GSE50760
DEG R-S	30	GBM	1.00 ± 0.00	0.89
DEG DB-S	28	GBM	1.00 ± 0.00	0.95
Var R-S	4	GLM	0.88 ± 0.14	0.89
Var DB-S	6	GBM	0.88 ± 0.15	0.89

Subsetting was performed based on the four HEFS stable signatures presented in this study. GBM: Gradient Boosting Machine. GLM: Generalized Linear Model.

The best model for each of the four scenarios was then evaluated on our external dataset, *i.e*. the GSE50760 dataset (see *Materials and methods*, *data collection and processing* section). As for the TCGA dataset, we retrieved four subsets from the evaluation dataset with different sets of variables corresponding to the four stable HEFS signatures. The evaluation on the GSE50760 subsets resulted in high MCC scores, i.e. 0.95 when using the DEG DB-S signature and 0.89 in the other three cases. Though all four signatures (both the short Var and the long DEG-based) managed to successfully separate Stage IV and Normal samples, features do not have the same importance in the decision process. Interestingly, the most informative seem to be the three variables that are common to the four scenarios: OTOP2, ETV4 and ESM1 (see Supplementary Figure S5).

The shortest stable signature producing good separation is the one from the Var R-S scenario that is composed of four genes: OTOP2, AJUBA, ESM1 and ETV4. AJUBA is the only gene that was not found in DEG-based HEFS scenarios. In (62) OTOP2 was cited as a potential biomarker of the CRC. OTOP2 was proved to be involved in proton channel activity, which is consistent with our biological problem of interest as the colorectal cancer disease is closely related to electrolyte changes (63). ETV4 has been suggested as a potential therapeutic target in CRC context in (64,65). ESM1 promotes CRC progression, cell migration and invasion (66) and might be of interest as a therapeutic target (67). From the four submitted genes, the ToppGene tool for gene list enrichment analysis found ETV1 and ESM1 to be significatively associated (*P*-adjusted by Bonferroni is 3.348 × 10^–2^) to the PD-0325901 drug (also known as mirdametinib). This drug has been investigated in CRC context in two clinical trials, NCT00147550 and NCT02510001. Finally, AJUBA was found as being associated with cell migration in colorectal cancer (68,69).

### Aggregation method evaluation on CRC dataset

**Figure 6.**
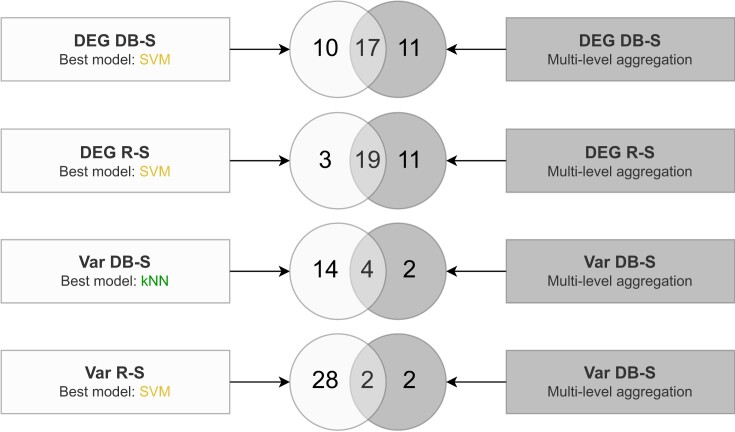
Comparison of signature composition between a standard feature selection strategy (light gray) and the hybrid ensemble feature selection approach presented in this paper (dark gray). Comparisons are displayed based on four possible scenarios including a filtering step by differentially expressed genes analysis (DEG) or variance (Var), followed by a 10 folds repeated holdout sampling either by random stratification (R-S) or distribution-balanced stratification (DB-S).

Here we analyze the benefits of our multi-level aggregation method by comparing it with a classical feature selection procedure (Figure 6). This procedure consists in taking the best performing model over thousands of trained ones regardless of the sampling run. For each of our four scenarios, we selected the best model with the best AVG MCC as well as the best STD MCC and other performance metrics, such as the accuracy, across ML methods and samplings. We then compared these four model signatures to the ones output by our four HEFS scenarios. Overall, SVMs models were found to be the best, with the exception of the Var DB-S scenario for which a kNN model outperforms them.

As a reminder, features that are selected within each stable HEFS signature have to be selected for at least 50% of samplings and by at least one model of each classifier. Thus, when analyzing the DEG DB-S scenarios, there are more compelling arguments in favor of the addition of 11 and 17 genes as opposed to the 10 genes given by the single SVM model. Indeed, regarding feature selection stability with the Nogueira score (Figure 5.B), SVM models are among the classifiers with the lowest scores for all scenarios and with a value of 0.72 in particular for both DEG scenarios. It highlights the great instability of the SVM model signatures between them compared to model signatures of other types of classifiers. Moreover, the signature lengths for SVM models are larger, regardless of the scenarios (Figure 5A). These results demonstrate the tendency of SVM to select longer signatures with a variable composition of features. Compared to the 10 or 3 specific features identified by the SVM models in DEG scenarios, the 11 additional variables that are specific to HEFS signatures offer the guarantee of having been obtained by at least 5 SVM models through the 10 sampling runs in addition to other types of ML models.

These observations can be extended to the Var scenarios. Indeed, the best models in those scenarios compute long signatures from which, an important part is not robust as it does not overlap with HEFS results (14 out of 16 for Var DB-S and 28 out of 30 for Var R-S). In this case it becomes even more important to integrate data and functional perturbations to identify the most stable variables while filtering the dataset based on variance.

### Exploring the generalization of the HEFS approach using a light mode

In this section we investigate the ability of our approach to generalize to other cancer use cases for retrieving stable variables along the aggregation process. To speed up the process, this study was conducted using a light mode of the HEFS approach. Compared to the standard HEFS mode, the number of trained wrappers was reduced from an average of 88 000 per scenario to 38 990, by eliminating highly costly resource-intensive algorithms and parameters, such as Bayes Networks or certain SVM kernels (Supplementary Table S2 for an overview of included algorithms and parameters), which provided minimal improvement compared to other methods and were computationally expensive. Moreover, for the light mode we exclusively utilize the DB-S sampling method since no significant differences were observed in the CRC Stage IV dataset between the DB-S and R-S samplings.

Finally, to be less stringent and allow more variability to be taken into account, we relaxed the threshold for the number of sampling runs to 40% instead of 50% (see *Materials and methods*, *wrapping and aggregation* section).

The light mode HEFS procedure was used to compute stable signatures for identifying biomarkers that distinguish KIRC Stage I samples from Normal samples, LUAD Stage I samples from Normal samples and UCEC Stage III samples from Normal ones. Kidney Renal Clear Cell Carcinoma (KIRC) is indeed one of the deadliest cancers with a high rate of patients belatedly diagnosed with metastasis (70), while Lung Adenocarcinoma (LUAD) counts as a large part of overall lung cancers that are still highly diagnosed every year (71). Identifying Stage I KIRC and LUAD biomarkers is an important task as the early detection of these cancers remains challenging. Finding late stage biomarkers of Uterine Corpus Endometrial Carcinoma (UCEC) is also of great interest as it is the most common cancer of the female reproductive system and is increasingly diagnosed and associated with a decrease of the age at which it occurs (72). To ensure the robustness of the light mode with respect to the standard mode, the light mode approach was further validated by re-analyzing the colorectal cancer (CRC) dataset.

After processing the data, we obtained 69 Normal and 262 Stage I samples for the KIRC dataset, 45 Normal and 186 Stage I samples for the LUAD one, and 19 Normal and 108 Stage III samples for the UCEC dataset. For the CRC cohort 51 Normal and 87 Stage IV samples were gathered as detailed above. Notably, the KIRC, LUAD and UCEC cohorts show a ratio of about 1:5 Normal versus Stage I/III samples, where CRC exhibits a 1:3 ratio of Normal versus Stage IV samples.

Notably, the results of the HEFS procedure on the CRC dataset are comparable between the light and standard modes. The Var filtering in the light mode retained the same 4 469 variables as in the standard mode, while the DEG filtering of the light mode retained 6 825 variables. In the HEFS light mode, 33 and 5 genes were respectively selected for the DEG DB-S and Var DB-S signatures, with two variables not overlapping (AJUBA and CPNE7) as they are specific to Var scenario. AJUBA was already the only gene that was specific to Var compared to DEG in the standard HEFS mode. Significantly, 4 of the genes of Var DB-S light mode are included in the 6-variable long signature of the Var DB-S standard mode described in the previous sections. Additionally, 25 out of the 33 genes identified for the DEG DB-S light mode are in common with the DEG DB-S standard mode. Three genes from the DEG DB-S standard mode signature were not selected in the light mode. However, these genes were among the less stable ones in the standard mode, as they were only selected in five out of the ten samplings at the last aggregation step.

**Figure 7.**
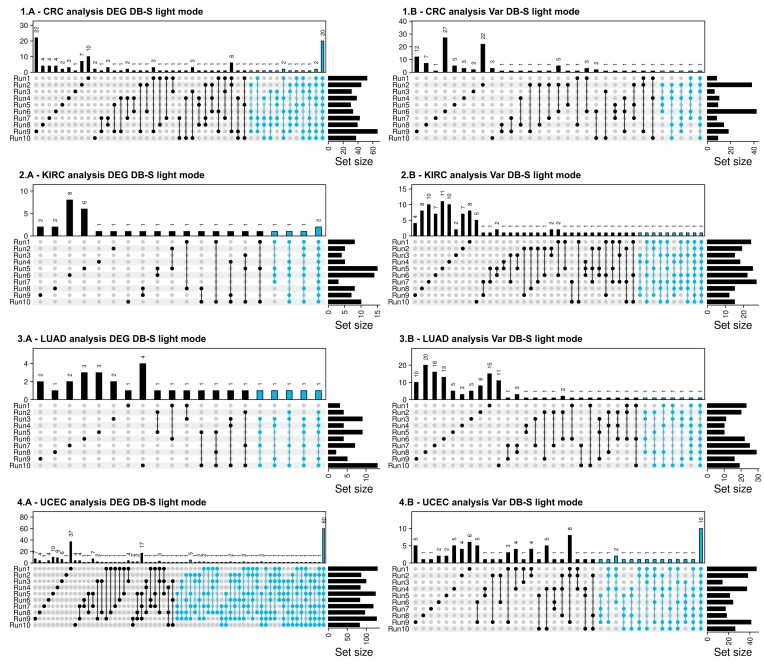
Feature distribution through all sampling runs for the 2 light HEFS scenarios (DEG DB-S and Var DB-S) based on the feature lists obtained after the second aggregation step, *i.e*. sampling-wise and for the four cancer use cases (1A–B: CRC Normal versus Stage IV, 2A–B: KIRC Normal versus Stage I, 3A–B: LUAD Normal versus Stage I and 4A–B: UCEC Normal versus Stage III). Features in blue are the ones kept in the last aggregation step and considered as a stable signature.

For the KIRC case, the signatures of the two light HEFS scenarios (DEG DB-S and Var DB-S) are 5-variable long (ATP6V0A4, TMEM213, ATP6V0D2, IRX2, SLC9A4) and 10-variable long (ADAM18, AC073172.1, SEMG2, LINC01983, LINC02437, PRR35, LINC00864, AC090709.1, LINC02121, AL160286.3) for DEG DB-S and Var DB-S respectively (Figure 7). After filtering, 5 353 variables were kept for Var and 7 891 for DEG, with a 32% overlap. The signatures are distinct, but the genes composing them are among the 32% common variables. The DEG DB-S signature contains genes already known to be involved in KIRC, while the Var DB-S signature includes less studied long non-coding RNAs (Supplementary Table S3).

For the LUAD case, the DEG DB-S in light mode successfully computed a 5-variable long signature (EMP2, AGER, PDLIM2, STX11, GYPE), while the Var DB-S light mode computed a 7-variable long one (EMP2, AGER, SGCG, PTPN21, STX11, GYPE, LGR4) (Figure 7). The two signatures show an overlap of four genes (EMP2, AGER, STX11, GYPE) with two of them known to be linked to lung carcinoma (Supplementary Table S3).

Finally, for the UCEC case, the DEG DB-S light mode resulted in a 102-variable long signature and the Var DB-S light one computed a signature of 24 variables. These two signatures have an overlap of 16 genes meaning that a third of the Var signature is included in the DEG one. Both signatures include highly interesting genes in the DisGeNet Endometrial Carcinoma annotation (Supplementary Table S3).

## Discussion

Feature selection (FS) is a key task in the identification of meaningful patterns through large-scale datasets. Hybrid Ensemble Feature Selection (HEFS) approaches are increasingly developed in this direction by taking advantage of multiple types of perturbations while ensuring the generalization of the selection outcome. Here we performed an extensive analysis of four types of HEFS scenarios to investigate transcriptomic data for the identification of biomarkers in Stage IV CRC, Stage I KIRC, Stage I LUAD and Stage III UCEC. We discuss the different choices that have to be made in the HEFS process and their impact on the final signature and on the classification result.

First, a crucial step in FS is to reduce the number of dimensions by filtering out the variables least likely to carry meaningful information. In a transcriptomic context, researchers classically apply a DEG filter before using ML approaches. However, one should be aware that DEG based filtering introduces bias as it takes into account the class of samples to achieve its analysis which is counter-intuitive for the subsequent use of ML. Indeed, a ML process requires computation of a train and a test set, while it is supposed to be blind to the class information during the training part. Another solution, as exposed in Bommert et al. (20), relies on a Var filter in gene expression survival data. Here we applied the classical Var filter without any *a priori* for the class in the context of cancer diagnosis biomarker identification from expression data, using HEFS approaches. In this study we extensively analyzed our standard HEFS scenarios on the CRC dataset. We observe that ML models trained on DEG filtered datasets were over-optimistic (AVG MCC score close to 1 and associated STD MCC close to 0), with long redundant signatures (24 to 38 variables in most cases), which are classical indications of overfitting. On the other hand, ML models trained on Var filter based datasets were slightly less efficient but still gave high quality results (AVG MCC around 0.8) and with shorter signatures (from 7 to 17 variables in most cases) as the input dataset was noisier. The variables computed for the Var scenarios on the CRC dataset were predominantly found in the DEG signatures. This pattern was also observed when computing a Stage I LUAD and a Stage III UCEC biomarker signature using the light mode HEFS.

It is important to keep in mind that in ensemble feature selection (EFS) in general, and thus in Hybrid EFS (HEFS) in particular, one looks for a tradeoff between diversity and stability. DEG-based signatures tend to be highly stable through models of the same type of classifier but also between classifier types and between sampling runs. Indeed, at least 28 features were found to be of interest after the aggregation step in HEFS DEG-based standard scenarios. Var-based signatures on the other hand are more diverse while being able to aggregate and compute short, stable signatures. For the CRC dataset, the 4-feature long Var-based signature obtained with the HEFS standard mode was generalized to another Stage IV CRC dataset with a similar efficiency to the one obtained for the longer DEG signatures. Overall, while DEG is widely used to reduce dimensions, it has the disadvantage of being data type dependent and prone to overfitting due to the integration of data classes in its strategy. On the other hand, a Var based filter operates independently of the data type and does not presuppose any differences between the classes. However, it produces noisier training datasets and may overlook interesting varying genes when dealing with highly imbalanced classes. This can be partly overcome through HEFS strategies combining both data and functional perturbations, and by confronting several methods to maximize their advantages. For example, on the KIRK dataset, DEG and Var filters complement each other as DEG detects strong differential patterns, while Var reveals different, less apparent ones.

A second important parameter to consider in a HEFS procedure is the choice of the data perturbation strategy. Here, we were interested in a repeated holdout scheme with a standard random stratified sampling (R-S) compared to a less used distribution-balanced stratified sampling (DB-S). The latter is intended to catch and represent possible intra-class heterogeneity in the wrapping process of our HEFS approach. Based on our results, we detected no significant changes between R-S and DB-S based wrapping models and the quality of their associated gene signatures. This may suggest that either the intra-class heterogeneity was handled through the wrapping FS process, or that the intra-class effect in our datasets was too weak to have an impact. Either way, we believe that intra-class heterogeneity in the context of FS for cancer is something to be considered and that should be investigated more.

This study explores the use of a large-scale aggregation process to take advantage of a variety of combinations of data re-samplings and ML models. When testing multiple ML algorithms, researchers usually compute a performance metric and select the model giving the highest score on a standard sampling. However, scores can be extremely close to one another, while the associated model signatures can be highly different. Choosing a unique model in this context can lead to the selection of non-robust features, wasting valuable resources on their characterization. Additionally, it may result in overlooking additional signals and more robust features, thus delaying important clinical research. To overcome this variability we performed an extensive aggregation process by training different types of classifiers, with thousands of various hyper-parameter configurations and based on multiple sampling runs. We therefore ensure that the proposed stable signatures of our HEFS approaches are not dependent on a unique ML model, type of classifier, performance ranking or data subset. Results on our datasets suggest that this type of aggregation produces stable signatures that can be generalized to other datasets.

Our aggregation approach successfully generalized by computing stable signatures for the characterization of Stage I KIRC, Stage I LUAD and Stage III UCEC. The HEFS approach in light mode computed stable signatures for DEG DB-S and Var DB-S for all cancer cases. The absence of overlap between the two signatures of Stage I KIRC can be explained by the ML algorithm's ability to decipher alternative strong patterns from different sets of variables and samplings. This finding underscores the complementarity of the two scenarios and emphasizes the importance of the filtering step that greatly impacts the rest of the process.

Finally, we addressed the computational cost of the HEFS by implementing a light mode. This mode was able to decipher stable signatures on the Stage IV CRC use case in significantly less time than the standard mode, completing the task in just 1 day compared to the previous 7 days required for parallel training of wrapper types (using SLURM jobs limited to 150G memory and 10cpus). Furthermore, on the CRC dataset, signatures in DEG DB-S and Var DB-S light mode scenarios were found to be highly similar to those in the standard mode, demonstrating the effectiveness and reliability of our light mode approach. An extensive approach is recommended to ensure stability across a broad spectrum of algorithms. However, a light version could be adequate to compute a minimally robust and more cost-effective biomarker signature.

## Conclusion

The work presented in this paper is a step towards the computation of robust biomarker signatures from transcriptomic data in a cancer context, by addressing the broad question of how to design hybrid ensemble feature selection (HEFS) approaches. More precisely, we were interested in exploring three crucial conception steps: dimension reduction, data perturbation by data re-sampling and functional perturbation by multi-wrappers. To investigate these questions we implemented a multi-level strategy. First, we integrated a particular dimension reduction step by differentially expressed genes (DEG) analysis or by variance (Var) analysis. Then we performed two types of sampling: a standard one (stratified random sampling R-S) and a second one focusing on capturing intra-class variability (distribution-balanced sampling DB-S). To follow, we employed a large-scale wrapping process based on eight state-of-the-art classifiers with numerous hyper-parameter configurations. Finally, a multi-level aggregation scheme was designed to make use of all the data and functional variability and applied in order to compute stable biomarker signatures. This HEFS approach gave promising results for Stage IV colorectal cancer as the computed signatures were robust when confronted to an external dataset and in comparison with a standard feature selection process. Moreover, the approach was able to generalize in light mode on the same dataset and with three other datasets, Stage I kidney cancer, Stage I lung cancer and Stage III endometrial cancer. In conclusion, HEFS is a recommended approach for identifying complex disease biomarkers as its results are independent of any specific feature selection method or sampling structure. However, we emphasize here the need for careful attention to several key steps such as early dimensionality reduction, selection of an appropriate sampling strategy and integration of the intra-variability of multiple feature selection methods based on machine learning.

## Supplementary Material

lqae079_Supplemental_Files

## Data Availability

The data underlying this article were accessed from the GDC portal under the COAD, READ, KIRC, LUAD and UCEC IDs for TCGA cohorts and from the GEO portal under the GSE50760 ID for the evaluation cohort. Derived data are available at https://doi.org/10.5281/zenodo.11373866. A two steps pipeline to perform our HEFS approach from sampling to wrapping and aggregation steps is available at https://doi.org/10.5281/zenodo.10519180.
