# Supplementary material for: Optimizing hybrid ensemble feature selection strategies for transcriptomic biomarker discovery in complex diseases
Source: NAR Genom Bioinform. 2024 Jul 11;6(3):lqae079. doi: 10.1093/nargab/lqae079 (PMC11237901; doi:10.1093/nargab/lqae079)
Supplement: lqae079_Supplemental_Files [file lqae079_supplemental_files.zip › Supplementary_Data.docx]

1. **ML classifier hyper-parameters for HEFS standard mode**

| BAYES | A1DE  weka : Bayes.AveragedNDependenceEstimators.A1DE | -F  -M  -W  -S | [1, 2, 3, 4, 5, 6, 7, 8, 9, 10]  [0.1, 0.2, 0.5, 1, 2, 3, 4, 5]  True/False  [10, 20, 30, 40, 50, 60, 70, 80, 90, 100, 1120, 120, 130, 140, 150] |
| --- | --- | --- | --- |
|  | Bayes Network  weka: bayes.BayesNet | -D  -Q  -E | True/False  [default TAN,  HillClimber [-P 1, -P 2, -P 3, -P 100000, -P 1 -R, -P 2 -R, -P 3 -R, -P 100000 -R],  SimulatedAnnealing [-A 10 -D 0.001, -A 10 -D 0.5, -A 10 -D 0.999, -A 50 -D 0.001, -A 50 -D 0.5, -A 50 -D 0.999, -A 100 -D 0.001, -A 100 -D 0.5, -A 100 -D 0.999, -A 200 -D 0.001, -A 200 -D 0.5, -A 200 -D 0.999],  K2 [-P 1, -P 2, -P 3, -P 100000, -P 1 -R, -P 2 -R, -P 3 -R, -P 100000 -R]]  [BayesNetEstimator [-A 0, -A 0.5, -A 1],  BMAEstimator [-A 0, -A 0.5, -A 1, -A 0 -k2, -A 0.5 -k2, -A 1 -k2],  MultiNomialBMAEstimator [-A 0, -A 0.5, -A 1, -A 0 -k2, -A 0.5 -k2, -A 1 -k2],  SimpleEstimator [-A 0, -A 0.5, -A 1]] |
|  | Naive Bayes  weka: NaiveBayes | -K  -D | True/False  True/False |
| FUNCTION | SVM  weka: functions.SMO | -C  -N  -K | [0.001, 0.01, 0.1, 1, 10, 100]  [0, 1, 2]  [PolyKernel [-E 2, -E 3, -E 4, -E 5, -E10, -E20],  NormalizedPolyKernel [-E 2, -E 3, -E 4, -E 5, -E 10, -E 20],  RBFKernel [-G 0.01, -G 0.1, -G 1, -G 5, -G 10, -G 20, -G 30],  Puk [-S 0.01, -S 0.1, -S 1, -S 5, -S 5, -S 10, -S 20, -S 30]] |
| LAZY | kNN  weka: lazy.IBk | -K  -I  -F  -E  -X  -A | [1, 2, 3, 4, 5, 6, 7, 8, 9, 10, 11, 12, 13, 14, 15, 16, 17, 18, 19, 20]  True/False  True/False  True/False  True/False  [default LinearNNSearch, default KDTree, default FilteredNeighbourSearch, default CoverTree, default BallTree] |
| TREE | C4.5  weka: trees.J48 | -U  -O  -C  -M  -R  -N  -S  -Q | True/False  True/False  [0.25, 0.50, 0.75, False]  [0, 1, 2, 3, 4, 5, 6, 7, 8, 9, 10]  True/False  [2, 3, 4, 5, 6, 7, 8, 9, 10, False]  True/False  [1, False] |
|  | Random Forest  weka: RandomForest | -P  -I  -K  -M  -S  -N | [25, 50, 75, 100]  [100, 300, 500, 700, 900]  [0, 1, 2, 3, 4]  [0, 1, 2, 3, 4, 5, 6, 7, 8, 9, 10]  1  [0, 2, 3, 4, 5, 6, 7, 8, 9, 10] |
|  | Simple CART  weka: trees.SimpleCart | -S  -M  -N  -U  -H  -A  -C | 1  [0, 1, 2, 3, 4, 5, 6, 7, 8, 9, 10]  [2, 3, 4, 5, 6, 7, 8, 9, 10]  True/False  True/False  True/False  [0.1, 0.2, 0.3, 0.4, 0.5] |

Supplementary Table S1. Selected ML classifiers, their associated weka function name and chosen hyper-parameters grid.

1. **Clustering for DEG and Var scenarios**

**SupplementaryFigureS1.pdf**

Supplementary Figure S1. Hierarchical clustering of Stage IV samples from variance filtering TCGA CRC data using transcript expression and based on euclidean distance and Ward’s method for linkage. Patient samples are shown by the vertical view while transcripts compose the horizontal one. Two sample clusters are defined.

**SupplementaryFigureS2.pdf**

Supplementary Figure S2**.** Hierarchical clustering of Normal samples from variance filtering TCGA CRC data using transcript expression and based on euclidean distance and Ward’s method for linkage. Patient samples are shown by the vertical view while transcripts compose the horizontal one. Three sample clusters are defined.

**SupplementaryFigureS3.pdf**

Supplementary Figure S3. Hierarchical clustering of Stage IV samples from differentially expressed gene analysis filtering TCGA CRC data using transcript expression and based on euclidean distance and Ward’s method for linkage. Patient samples are shown by the vertical view while transcripts compose the horizontal one. Two sample clusters are defined.

**SupplementaryFigureS4.pdf**

Supplementary Figure S4. Hierarchical clustering of Normal samples from differentially expressed gene analysis filtering TCGA CRC data using transcript expression and based on euclidean distance and Ward’s method for linkage. Patient samples are shown by the vertical view while transcripts compose the horizontal one. Two sample clusters are defined.

1. **Feature importance for the 4 optimized models**

**SupplementaryFigureS5.pdf**

Supplementary Figure S5. Scaled importance of variables in each of the 4 optimized machine learning models trained on TCGA colorectal cancer data using the h2o R package. The TCGA dataset was subset based on the 4 different hybrid ensemble feature selection scenarios presented in the paper (DEG DB-S, DEG R-S, Var DB-S and Var R-S). The most important feature for a model has a score of 1 and the other scores are scaled and ranked to it. A score above 0 is shown by a bar, a score of 0 is highlighted by a 0 while the absence of the variable for the considered model is depicted with an X mark.

1. **ML classifier hyper-parameters for HEFS light mode**

| BAYES | A1DE  weka : Bayes.AveragedNDependenceEstimators.A1DE | -F  -M  -W  -S | [1, 2, 3, 4, 5, 6, 7, 8, 9, 10]  [0.1, 0.2, 0.5, 1, 2, 3, 4, 5]  True/False  [10, 20, 30, 40, 50, 60, 70, 80, 90, 100, 1120, 120, 130, 140, 150] |
| --- | --- | --- | --- |
|  | Naive Bayes  weka: NaiveBayes | -K  -D | True/False  True/False |
| FUNCTION | SVM  weka: functions.SMO | -C  -N  -K | [0.001, 0.01, 0.1, 1, 10, 100]  [0, 1, 2]  [NormalizedPolyKernel [-E 2, -E 3, -E 4, -E 5, -E 10, -E 20],  RBFKernel [-G 0.01, -G 0.1, -G 1, -G 5, -G 10, -G 20, -G 30],  Puk [-S 0.01, -S 0.1, -S 1, -S 5, -S 5, -S 10, -S 20, -S 30]] |
| TREE | C4.5  weka: trees.J48 | -U  -O  -C  -M  -R  -N  -S  -Q | True/False  True/False  [0.25, 0.50, 0.75, False]  [0, 1, 2, 3, 4, 5, 6, 7, 8, 9, 10]  True/False  [2, 3, 4, 5, 6, 7, 8, 9, 10, False]  True/False  [1, False] |
|  | Random Forest  weka: RandomForest | -P  -I  -K  -M  -S  -N | [ 50, 100]  [100, 300, 500]  [0, 1, 2, 3, 4]  [0, 2, 4, 6, 8, 10]  1  [0, 2, 4, 6, 8, 10] |

Supplementary Table S2. Selected machine learning classifiers for the light mode Hybrid Ensemble Feature Selection approach, their associated weka function name and chosen hyper-parameters grid.

1. **Disease annotation analysis of CRC, KIRC, LUAD and UCEC study cases by HEFS light mode**

| Phenotype | Scenario | Stable signature | DisGeNet enriched annotation analysis with adjusted p-value |
| --- | --- | --- | --- |
| CRC - Normal vs Stage IV | DEG DB-S | **CDH3**  **IL6R**  **SCGN**  **SALL4**  **ESM1**  **SCARA5**  **ETV4**  **OTOP2**  **PVT1**  **CBX8**  **EIF4E3**  **ABCA8**  **MRGBP**  **LYVE1**  GLP2R  BEST4  FAM135B  CLEC3B  ENPP6  KRT80  CA7  METTL7A  SLC39A10  ELANE  SEMA6A-AS2  MAMDC2  SLC51B  PLPP1  NKRF  VSTM2A  GLIPR2  UGP2  TMIGD1 | Colorectal cancer:  $1.2\times{10}^{-1}$ |
|  | Var DB-S | **OTOP2**  **AJUBA**  **ETV4**  KRT80  CPNE7 | Colorectal cancer:  $1.4\times{10}^{-1}$ |
| KIRC - Normal vs Stage I | DEG DB-S | **ATP6V0A4**  **TMEM213**  ATP6V0D2  IRX2  SLC9A4 | Conventional (Clear Cell) Renal Cell Carcinoma:  $5.6\times{10}^{-2}$ |
|  | Var DB-S | ADAM18  AC073172.1  SEMG2  LINC01983  LINC02437  PRR35  LINC00864  AC090709.1  LINC02121  AL160286.3 | Conventional (Clear Cell) Renal Cell Carcinoma:  NA |
| LUAD - Normal vs Stage I | DEG DB-S | **EMP2**  **AGER**  PDLIM2  STX11  GYPE | Carcinoma of lung:  $1.7\times{10}^{-1}$ |
|  | Var DB-S | **EMP2**  **AGER**  **SGCG**  PTPN21  STX11  GYPE  LGR4 | Carcinoma of lung:  $9.3\times{10}^{-2}$ |
| UCEC - Normal vs Stage III | DEG DB-S | **AURKA**  **DNMT3B**  **BIRC5**  **MYBL2**  **EZH2**  **CCNB1**  **CDC25C**  **PLK1**  **AURKB**  **CCNE1**  **CHEK1**  **FEN1**  **UHRF1**  E2F2  TACC3  RAD51  MCM10  PRR11  GTSE1  SPAG5  UBE2T  TPX2  CDC45  CBX7  CDKN3  GINS1  ASF1B  CDCA3  PLSCR4  CENPA  CDC20  NCAPH  ZWINT  HJURP  AUNIP  SGO1  CDCA8  TROAP  ESPL1  CENPO  KIF2C  NUF2  TBC1D7  MTFR2  CDCA5  CENPU  SKA1  CCNB2  TEDC2  CCNF  POC1A  PTTG1  MELK  SKA3  BUB1  SHCBP1  RRM2  UBE2C  KIF18B  JPT1  KIFC1  MEF2C-AS1  LINC02310  AP001528.3  ADH1B  CENPN  AC027449.1  KNSTRN  TICRR  PGD  AC107959.1  SPC24  FAXDC2  ORC1  HAPLN1  MAOB  CDC6  ADM2  RERG  SPC25  PCLAF  FAM83D  KLHDC1  CENPF  EME1  HMGB3  POLQ  ORC6  GPRASP1  PGM5P4  TTK  CKS2  DLGAP5  PIMREG  KIF11  ZNF25  KPNA2  CDCA2  ARHGAP11A  EFNA3  CEP55  AC004554.2 | Endometrial carcinoma:  $5.8\times{10}^{-3}$ |
|  | Var DB-S | **CCNB1**  **BIRC5**  **MYBL2**  **CDC25C**  ZWINT  STIL  CKS2  SKA1  TEDC2  UBE2C  IQGAP3  MEF2C-AS1  ASF1B  ZNF300P1  KIF20A  CDC20  HJURP  TCEAL6  TACC3  DEPDC1  TTK  TROAP  KLHL4  ASPA | Endometrial carcinoma:  $1.4\times{10}^{-1}$ |

Supplementary Table S3. DisGeNet annotation enrichment analysis based on the stable signature computed by our HEFS light mode in Var DB-S and DEG DB-S scenarios for the characterization of Stage IV Colorectal cancer (CRC), Stage I Kidney Renal Clear Cell (KIRC), Stage I Lung Adenocarcinoma (LUAD) and Stage III Uterine Corpus Endometrial Carcinoma (UCEC). Genes in bold are the ones involved in the associated DisGeNet annotation.
