# Supplementary figures and images for: Optimizing hybrid ensemble feature selection strategies for transcriptomic biomarker discovery in complex diseases
Source: NAR Genom Bioinform. 2024 Jul 11;6(3):lqae079. doi: 10.1093/nargab/lqae079 (PMC11237901; doi:10.1093/nargab/lqae079)

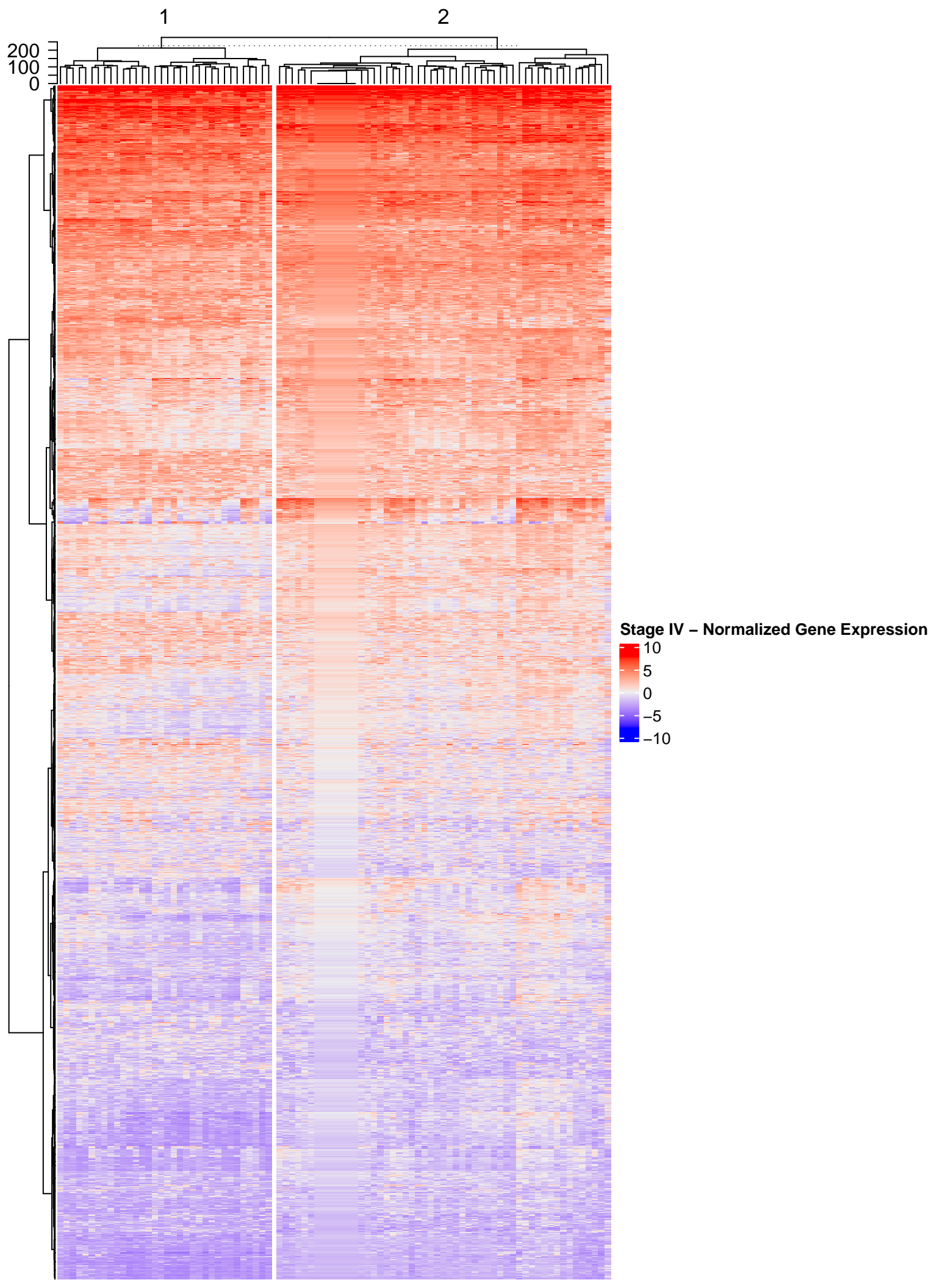

Supplement: lqae079_Supplemental_Files [file lqae079_supplemental_files.zip › SupplementaryFigureS1.pdf]

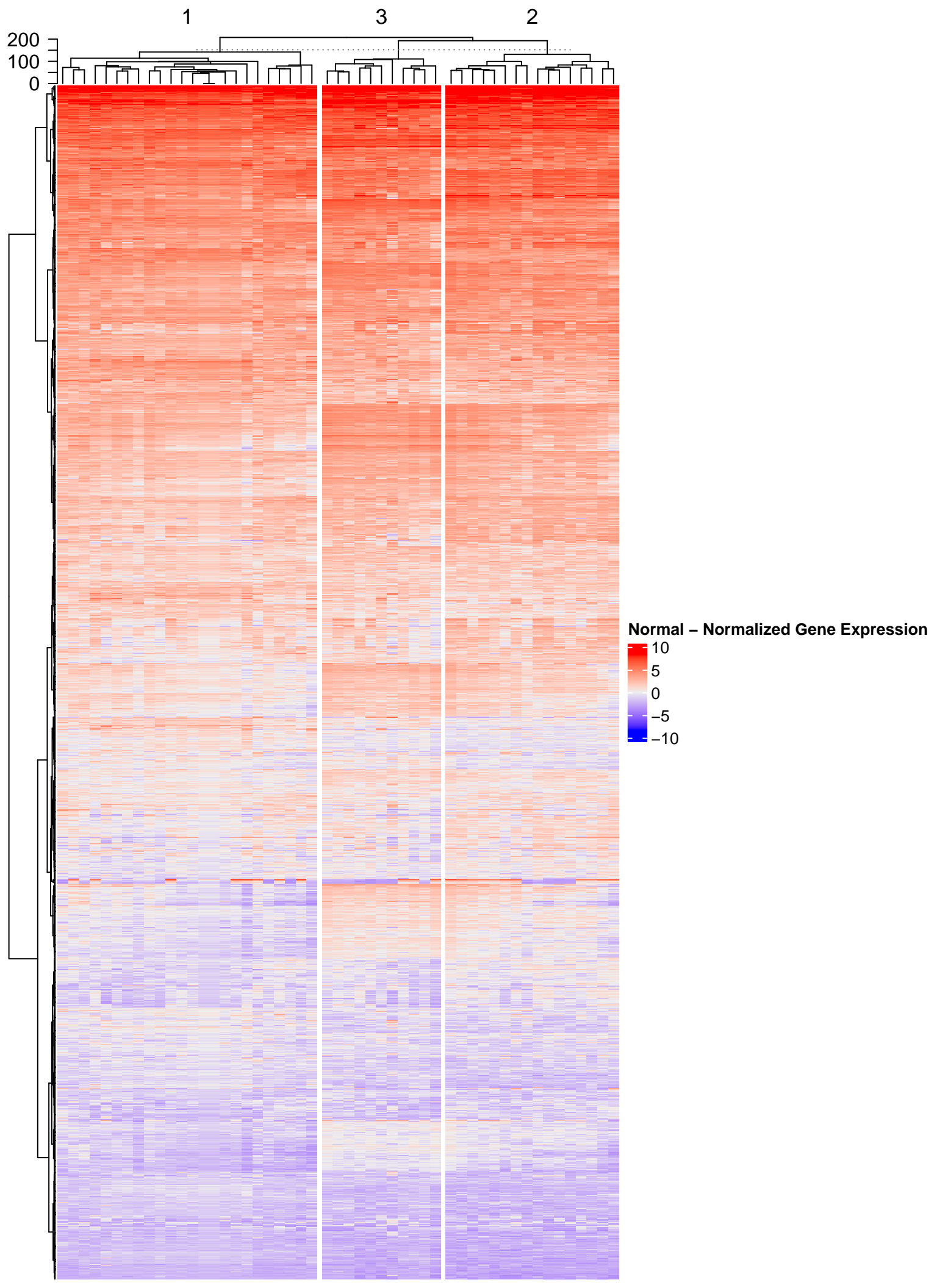

Supplement: lqae079_Supplemental_Files [file lqae079_supplemental_files.zip › SupplementaryFigureS2.pdf]

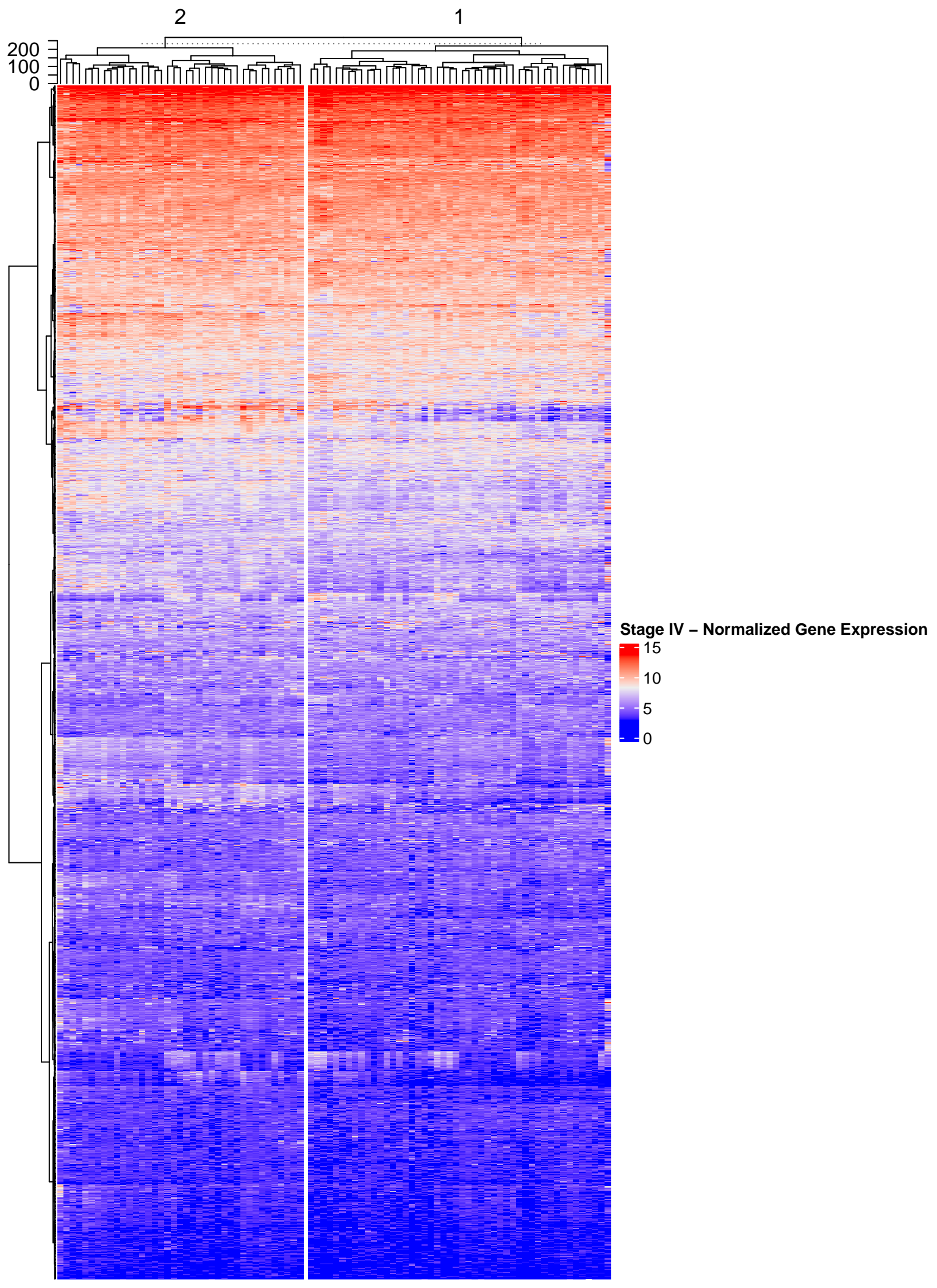

Supplement: lqae079_Supplemental_Files [file lqae079_supplemental_files.zip › SupplementaryFigureS3.pdf]

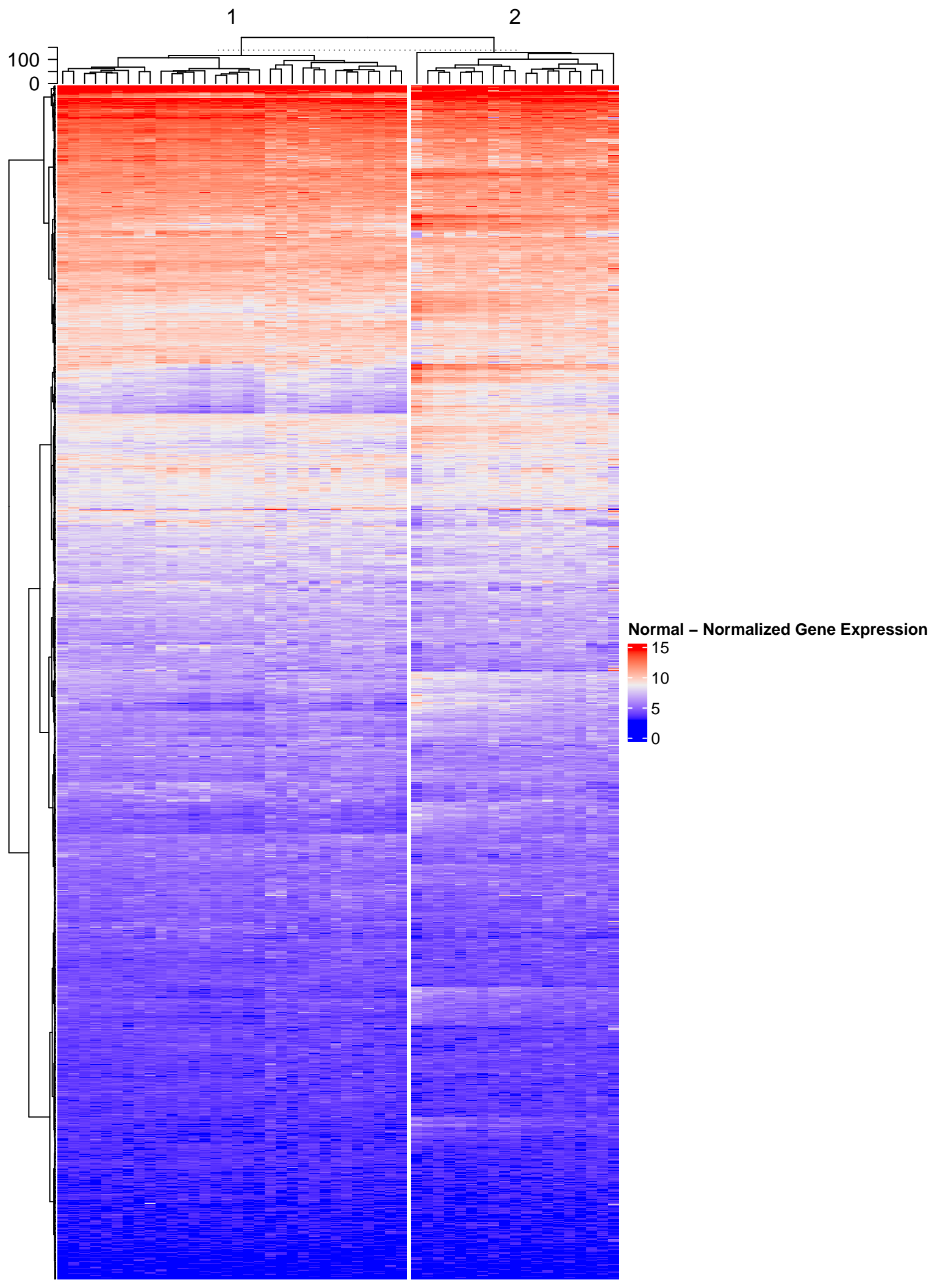

Supplement: lqae079_Supplemental_Files [file lqae079_supplemental_files.zip › SupplementaryFigureS4.pdf]

Variable

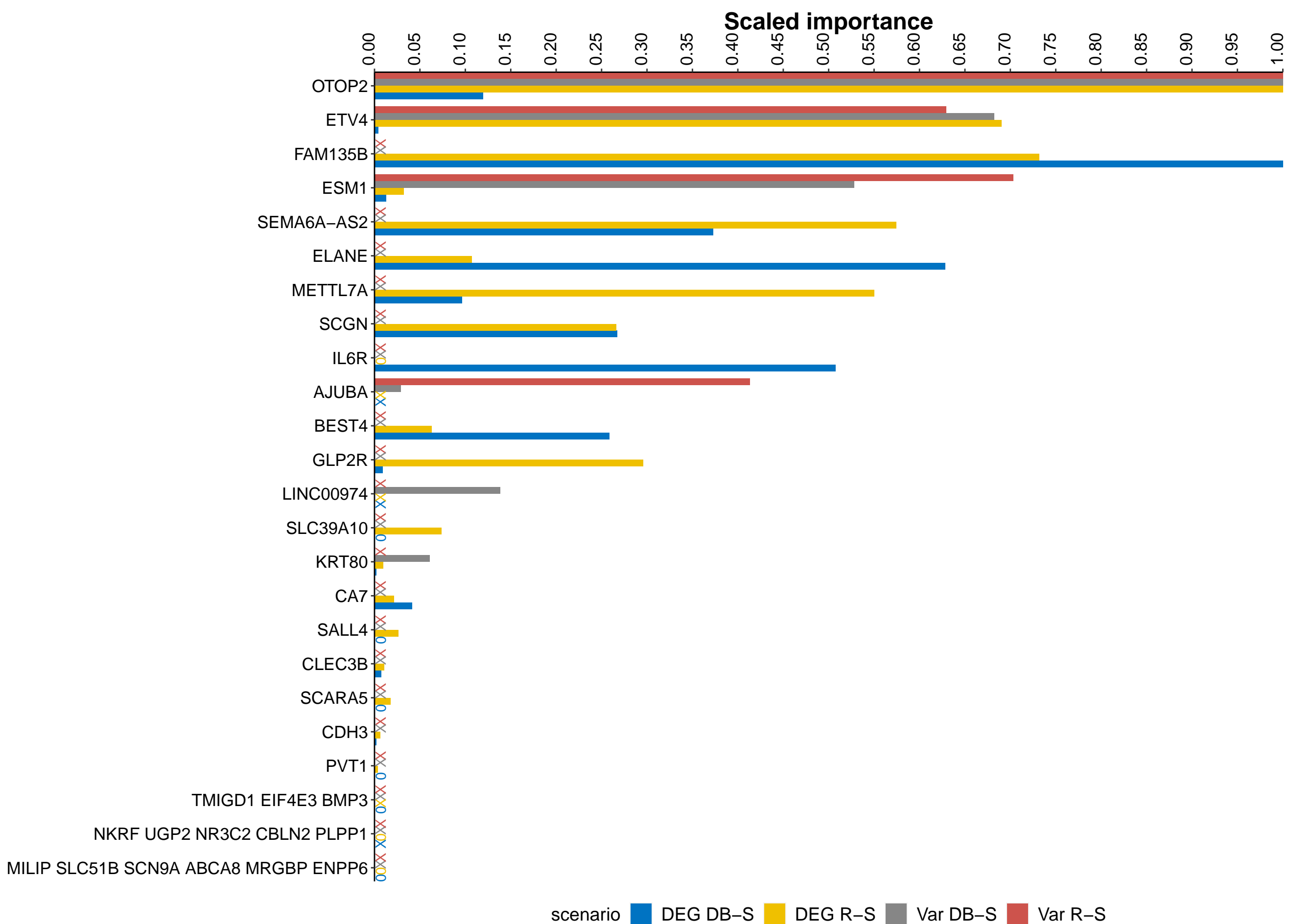

Supplement: lqae079_Supplemental_Files [file lqae079_supplemental_files.zip › SupplementaryFigureS5.pdf]
